# The Effect of the Salt Water Aging on the Mechanical Properties of Epoxy Adhesives Compounds

**DOI:** 10.3390/polym12040843

**Published:** 2020-04-06

**Authors:** Anna Rudawska

**Affiliations:** Faculty of Mechanical Engineering, Lublin University of Technology, Nadbystrzycka 36 Str., 20-618 Lublin, Poland; a.rudawska@pollub.pl; Tel.: +48-81-538-4232

**Keywords:** epoxy adhesive compounds, aging, seawater, mechanical properties

## Abstract

The objective of this study is to compare the effect of selected operating factors on the mechanical properties of epoxy adhesive compounds aged in salt water. Five different water environments were tested: tap water, normal seawater (reference salinity value), seawater with double reference salinity value, seawater with half of the reference salinity and seawater with a quarter of the reference salinity value. Samples of two different adhesive compounds were prepared using the epoxy resin and triethylenetetramine curing agent. One of the compounds was filled with calcium carbonate. The samples were aged in five different water environments for three months, one month and one week, respectively. Mechanical properties of the cured adhesive compound samples were determined via strength tests performed on the Zwick/Roell Z150 testing machine in compliance with the EN ISO 604 standard. The objective of the experiments was to determine the effect of different seawater environments on selected mechanical properties (including strength) of the fabricated adhesive compounds.

## 1. Introduction

Properties of materials are of crucial importance in engineering. They play a fundamental role in the selection of elements for different types of structures and machines. These components should have very high fatigue strength and resistance to various environments in which they will be used. For this reason, researchers analyze all possible solutions before introducing something completely new.

Epoxy-resin-based adhesive compounds are used not only for manufacturing elements but also for joining them [1,2,3,4]. These compounds are applied in many branches of industry, predominantly in the aviation and marine industries. In the aviation industry, they are used to join components and thus create structures that are much lighter than welded joints. In the marine industry, on the other hand, they are used for coating and laminating wooden boat structures against seawater corrosion, among others. Epoxy compounds have very good mechanical properties such as strength and rigidity. They have high resistance to elevated temperatures, and some of them are also fireproof. In liquid form, they can be used for joining elements made of different materials. When subjected to curing, however, these materials form solid, resistant structures with low specific weight, which show different properties in various environments [5,6,7,8,9], including water [10,11,12,13], moisture [14,15,16], humid [17,18,19] or hydrothermal conditions [20].

As already mentioned, epoxy adhesives and coatings used in the marine industry are often exposed to the impact of seawater, one of the most harmful environments affecting the service life of engineering structures. Diverse aspects of the seawater’s environmental effect on the strength of adhesive joints and adhesives were analyzed by different authors [21,22,23,24,25,26,27]. Fernades et al. [13] characterized the fracture envelope of a commercial epoxy adhesive used in the automotive industry as a function of the water content in the adhesive. The salt water and distilled environments were used as aging environments of DCB (Double Cantilever Beam) and ODCB (open-DCB) specimens. Bordes et al. [21] investigated the long-term behavior of adhesively bonded double lap shear steel joints aged in seawater and the degradation of mechanical properties of an epoxy adhesive. Aging on bulk adhesive was performed in deionized water, salt and seawater at three temperatures, 20, 40 and 60 °C. Heshmati et al. [15] investigated the effects of aging the epoxy adhesive and bonded joints (fiber-reinforced-polymer/steel joints) in five harsh environments, including salt water solution at various temperatures (20 °C and 45 °C). Three-dimensional (3D) moisture diffusion properties of different FRPs (fibre reinforced polymer/steel) and adhesive material in various aging conditions were characterized. In another work, Heshmati et al. [9] presented the effects of cyclic wet–dry, freeze–thaw and combined wet and freeze–thaw conditions (distilled water and salt water, when combined with freeze–thaw for 125 and 250 cycles) on the mechanical behavior of bonded FRP/steel joints. The results have shown, among other things, that the ductility of the adhesive is reduced after it has been dried from the wet state.

The objective of this study is to compare the selected mechanical properties of cured adhesive compounds (unmodified and modified alike) aged in seawater environments with different salt concentrations. The experiments aim to determine and compare the compressive strength, compression modulus and compressive strains of the cured epoxy adhesives under study.

## 2. Materials and Methods

### 2.1. Tested Epoxy Adhesive Compounds

Two variants of epoxy adhesive compounds were tested: unmodified and modified. Unmodified epoxy adhesive compounds were fabricated using two ingredients: epoxy resin (Epidian 53 (trade name), CIECH Resins, Nowa Sarzyna, Poland) and triethylenetetramine curing agent (Z-1 (trade name), CIECH Resins, Nowa Sarzyna, Poland). Modified epoxy adhesive compounds were prepared using three ingredients: epoxy resin (Epidian 53 (trade name), CIECH Resins, Nowa Sarzyna, Poland), triethylenetetramine curing agent (Z-1 (trade name), CIECH Resins, Nowa Sarzyna, Poland) and calcium carbonate (CaCO_3_).

#### 2.1.1. Epoxy Resin

Epoxy resin, trade name Epidian 53, is a styrene-modified epoxy resin. It is a compound consisting of a mixture of Bisphenol A and Epichlorohydrin, with an average molecular weight of less than 700 and with the addition of styrene. The mechanical properties of the cured Epidian 53 epoxy resin are given in Table 1.

The selected physicochemical properties of Epidian 53 epoxy resin were presented in [6].

#### 2.1.2. Curing Agents

Marketed under the trade name of Z-1, triethylenetetramine (CIECH Resins, Nowa Sarzyna, Poland) is an amino curing agent. It is predominantly used for curing low-molecule resin compounds and products based on such resins. It is widely used for curing liquid epoxy resins. The addition of Z-1 leads to the increased elasticity and impact strength of adhesives, hence it is used for fabricating adhesive-bonded joints that are exposed to deformation (e.g., rubber–metal joints) or for potting electronic components. The properties of the triethylenetetramine curing agent are given in Table 2 and in [7].

#### 2.1.3. Calcium Carbonate Modifying Filler

Calcium carbonate (structural formula: CaCO_3_) is an inorganic chemical compound also known as (IV) calcium carbonate. This solid substance has a density of 2.83 g/cm^3^ and usually comes in the form of fine white powder. It is water-insoluble and has a melting point of 825 °C. Calcium carbonate is commonly found in nature as the main component of minerals. In the natural environment, it is found in sedimentary rocks that are processed into powder, thus enabling further use of calcium carbonate. Calcium carbonate is also used as a filler for modifying epoxy resins. This inactive filler can modify adhesive compounds only to a small degree. The addition of calcium carbonate to an epoxy resin causes turbidity of the compound and relatively reduces the manufacturing costs of the adhesive. Nevertheless, in some cases, the addition of inactive fillers may deteriorate the properties of an adhesive and reduce its strength. Some information regarding this modifying agent was presented in [29].

### 2.2. Preparation of Epoxy Adhesive Compounds

Table 3 presents the two variants of the tested epoxy adhesive compounds containing the components described in Section 2.1. The epoxy resin and the curing agent were mixed in the stoichiometric ratio of 100:80 both in unmodified and modified epoxy adhesive compounds. In addition to the ingredients listed, modified epoxy adhesive compounds contained calcium carbonate in the amount of 2 g per 100 g epoxy resin and 80 g curing agent.

The epoxy adhesive compounds were prepared in several stages. First, the appropriate amounts of the epoxy adhesive compound components were weighed using the OX-8100 balance (FAWAG S.A, Lublin, Poland; reading accuracy: 0.1 g, ISO 9001). The first of the tested epoxy adhesive compounds was prepared by adding the curing agent to the resin, and mixing them in a mixing device with the use of a special disk mixer. The components were mechanically mixed in a polymer container for 90 s at a shear rate of 128 m/min until a homogeneous mass was obtained. Gas bubbles formed during mixing were removed from the compound with the use of a vacuum pump. The epoxy adhesive compound components were weighed and mixed at a temperature of 23 °C ± 3 °C and a humidity of 23% ± 6%.

The other of the tested epoxy adhesive compounds (the one filled with calcium carbonate) was prepared in the following way: first, appropriate amounts of the epoxy resin and the calcium carbonate filler were weighed (Table 3) and mixed. After that, the curing agent was added to the compound. The preparation process parameters for both modified and unmodified epoxy adhesive compounds were the same.

### 2.3. Shape, Dimensions and Fabrication of the Adhesive Compound Samples

**Figure 1:**
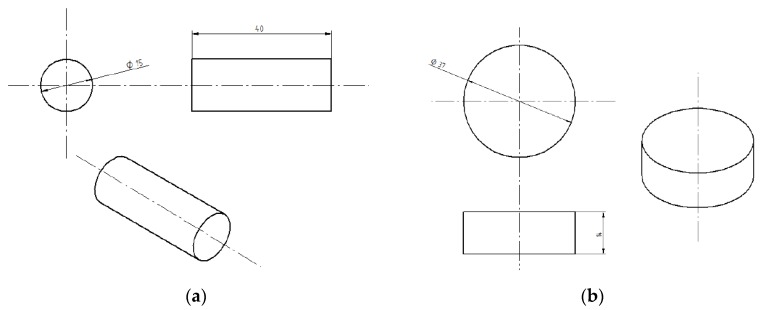
Shape and dimensions of adhesive compounds samples: (**a**) strength test samples; (**b**) microscopic test samples.

**Figure 2:**
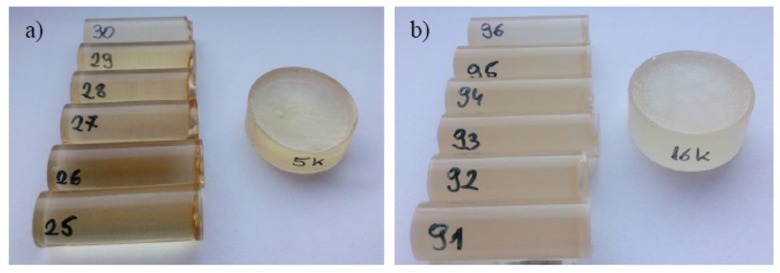
Real view of cured epoxy adhesive samples: (**a**) unmodified (Epidian 53/Z-1); (**b**) modified (Epidian 53/Z-1/CaCO_3_)

Two types of cylindrical samples were used in the experiments: strength test samples (Figure 1a) and microscopic test samples (Figure 1b). Figure 2 shows the real samples of the cured adhesive compounds (unmodified and modified).

The test samples were fabricated by casting with the use of 10 mL and 20 mL cylindrical molds. Following their preparation, the epoxy adhesive compounds (unmodified and modified alike) were poured into the molds coated inside with silicon spray (Polsiform, Polish Silicones, Poland) for an easy release. The anti-adhesion agent was applied by spraying from a distance of 100 mm for 1 min. The samples were left to cure (single-stage) for 7 days, at an ambient temperature of 2 °C ± 3 °C and a humidity of 23% ± 6%.

The samples were machined by grinding and milling in order to make them uniform in terms of length and surface smoothness. The finished samples (Figure 1a) had an average length of 40 ± 0.3 mm and an average diameter of 15 ± 0.1 mm. Next, the samples were marked with numbers and divided into batches of 12 pieces each. Each batch contained 6 samples of unmodified epoxy adhesive (Epidian 53/Z-1) and 6 samples of modified epoxy adhesive compound (Epidian 53/Z-1/CaCO_3_) (Figure 2). In total, 192 cylindrical samples (Figure 1a) were prepared for the purpose of the study, 96 of these samples were made of Epidian 53/Z-1, and the remaining 96 of them were made of Epidian 53/Z-1/CaCO_3_.

In addition, a total of 16 samples per each epoxy adhesive compounds variants (Table 3) were fabricated for subsequent microscopic tests (d = 37 ± 0.2 mm and l = 14 ± 0.2 mm, Figure 1b), of which 15 samples were subjected to the next stage of testing and one was left as a reference sample.

After the curing process, the samples were removed from the molds and conditioned for 24 h at an ambient temperature of 22 ± 1 °C and a humidity of 25% ± 1%. The samples were then placed in five different water environments (Table 4), with 19 samples per each of the tested environments described in Section 2.4.

### 2.4. Environment and Aging Time

The experiments involved subjecting the samples of the prepared epoxy adhesive compounds to aging in 5 different water environments, including tap water and 4 water environments with different salt concentrations (Table 4). The reference environment was seawater with a salinity of 35‰, which is the average salinity of the world’s seawater. Salt water concentration is expressed by salinity describing the average mineralization of seawater. Salinity is determined by measuring the grams of dissolved salts in 1 kg of seawater, excluding gases, suspensions and organic matter. On average, seawater in the world’s oceans has a salinity of about 35‰, which means that 1 kg of seawater has 35 g of NaCl [30].

Water environments were prepared by measuring out the appropriate portions of tap water and sea salt (NaCl). Room-temperature tap water was used in the tests. Depending on the tested water environment, the amount of sea salt was weighed out using the OX-8100 scales (FAWAG S.A, Lublin, Poland, weighing accuracy: 0.1 g, ISO 9001). NaCl was then mixed with tap water until complete dissolution. The mixing time varied depending on the salt content in the solution. The solutions were then kept in airtight glass containers. The experiments were conducted in three different time periods. The samples were divided into three batches, with each batch subjected to aging in the 5 tested water environments for 3 months, 1 month and 1 week, respectively.

### 2.5. Experimental Tests

The experiments involved two types of tests:

Strength tests that were performed by the destructive test method on scheduled times (depending on the aging period), in compliance with the PN-EN ISO 604 standard, using the Zwick/Roell 150 testing machine and the following parameters: initial load = 30 N, compression rate = 2 mm/min, test speed = 10 mm/min;

Microscopic tests that were performed on scheduled times (depending on the aging period), using the digital microscope (15×, 270× and 534× LED, Vitiny DIGI Microscope -UM06, Vitiny Producer, Taiwan) enabling the examination of the adhesive compounds’ structure and the impact of tested water environments on their appearance and properties.

The total amount of the tested epoxy adhesive samples amounted to 192 items (for each type of epoxy adhesive variants: unmodified and modified: 5 batches of environment types × 3 variants of aging time × 6 samples and 6 standard samples). The basic statistics of the results were considered. The mean and standard deviation were determined, rejecting the extreme values (gross errors) of the obtained results. Strength test results are compared depending on the seawater environment and aging time of the unmodified and modified adhesive compound samples.

## 3. Results

### 3.1. Strength Test Results

#### 3.1.1. Compressive Strength

**Figure 3:**
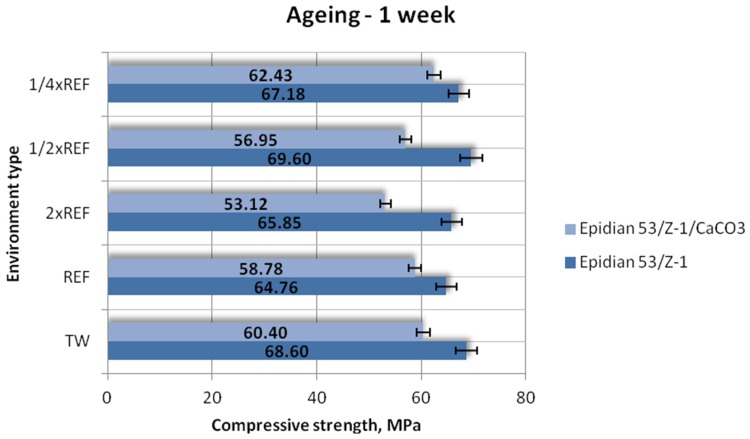
Compressive strength of the unmodified (Epidian 53/Z-1) and modified (Epidian 53/Z-1/CaCO_3_) epoxy adhesive compounds, aged for 1 week.

**Figure 4:**
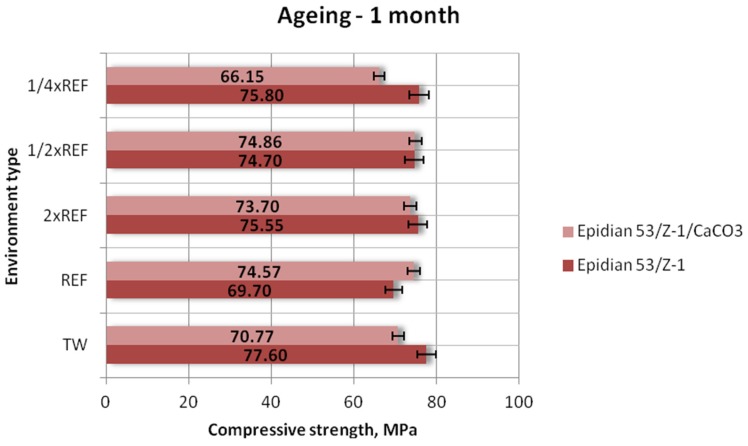
Compressive strength of the unmodified (Epidian 53/Z-1) and modified (Epidian 53/Z-1/CaCO_3_) adhesive compounds, aged for 1 month.

**Figure 5:**
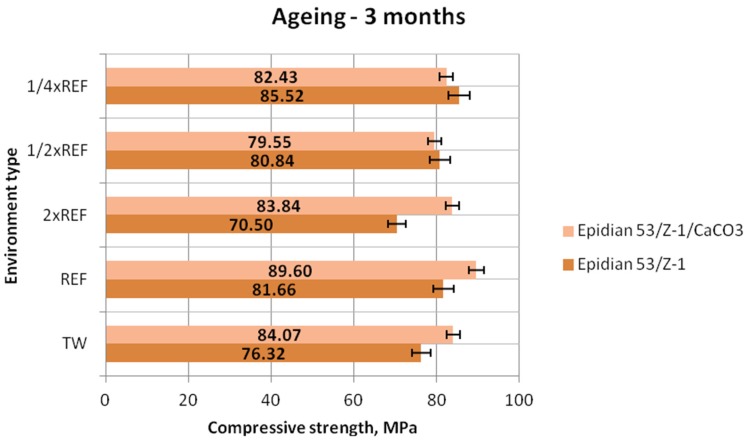
Compressive strength of the unmodified (Epidian 53/Z-1) and modified (Epidian 53/Z-1/CaCO_3_) adhesive compounds, aged for 3 months.

Figure 3, Figure 4 and Figure 5 show the results of compressive strength (mean values) of the two tested types of epoxy adhesive compounds: one unmodified and the other filled with calcium carbonate, aged in different seawater environments for one week, one month and three months, respectively.

The above results demonstrate that in all tested water environments, the Epidian 53/Z-1 samples have a much higher compressive strength than the samples of Epidian 53/Z-1/CaCO_3_. The difference between the highest compressive strength value of 69.60 MPa (1/2 reference salinity) and the lowest value of 64.76 MPa (reference salinity) is about 7%. The Epidian 53/Z-1/CaCO_3_ adhesive compound has a much lower compressive strength. The highest compressive strength equal to 62.43 MPa was obtained for the samples aged in the solution with a quarter of the reference salinity value (1/4×REF). The lowest compressive strength of 53.12 MPa was obtained for the samples aged in the seawater with double reference salinity (2×REF), this value amounting to 85% of the maximum value obtained during the strength tests. The compressive strength values obtained for other environments ranged from 56 MPa to 60 MPa. Based on the results, it can therefore be claimed that the compressive strength of the Epidian 53/Z-1/CaCO_3_ epoxy adhesive compound decreases with increasing the salt concentration.

As for the Epidian 53/Z-1 adhesive compound (Figure 4), the highest compressive strength was obtained for the samples aged in tap water and it was equal to 77.60 MPa. Compressive strength results of other samples are comparable: 75.80 MPa (1/4×REF), 75.55 MPa (2×REF (double reference salinity)) and 74.70 MPa (1/2×REF reference salinity). The samples aged in the reference seawater environment had the lowest compressive strength of 69.70 MPa, which amounted to 89% of the maximum achieved value. In the case of the adhesive compound filled with calcium carbonate, the highest compressive strength was obtained for the samples aged in the seawater environment with 1/2 reference salinity and it was equal to 74.86 MPa. The lowest compressive strength (66.15 MPa) was obtained for the samples aged in the solution with a quarter of the reference salinity value. Nevertheless, this value amounts to over 88% of the maximum achieved compressive strength value. The plot also reveals that in the environment with 1/2 reference salinity, both tested adhesive compounds exhibit almost identical compressive strength. In general, the results demonstrate that salt concentration does not have a significant impact on the compressive strength of the tested adhesive compounds. Nevertheless, higher values of this parameter were obtained for the Epidian 53/Z-1 samples.

The above results (Figure 5) demonstrate that the compressive strength of both adhesive compounds is similar. In the case of Epidian 53/Z-1/CaCO_3_, the highest compressive strength (89.60 MPa) was obtained for the samples aged in the reference seawater environment. A slightly lower value (84.07 MPa) was obtained for the samples aged in tap water, while the compressive strength of the Epidian 53/Z-1/CaCO_3_ samples aged in the environments with 2× reference salinity (83.84 MPa) and 1/4 reference salinity (82.43 MPa) is almost identical, amounting to approximately 93% of the maximum value obtained for this adhesive. The lowest compressive strength (79.55 MPa) was obtained by the samples aged in the environment with a quarter of the reference salinity value, and the compressive strength value differed only by 11% from the maximum achieved value. As for the unmodified epoxy adhesive compound, the highest compressive strength of 85.52 MPa was obtained for the samples aged in the environment containing a quarter of the reference salinity. An average strength of about 81 MPa was obtained for the samples aged in two environments: the reference environment (81.66 MPa) and that with 1/2 reference salinity (80.84 MPa). This value amounts to about 95% of the maximum achieved value.

The lowest compressive strength of 70.50 MPa was obtained for the Epidian 53/Z-1 samples aged in the environment with double reference salinity, which amounted to only 82% of the maximum achieved value. Comparing the results of these two adhesive compounds, it can be observed that the epoxy adhesive compound filled with calcium carbonate (CaCO_3_) has a higher compressive strength.

#### 3.1.2. Compression Modulus

**Figure 6:**
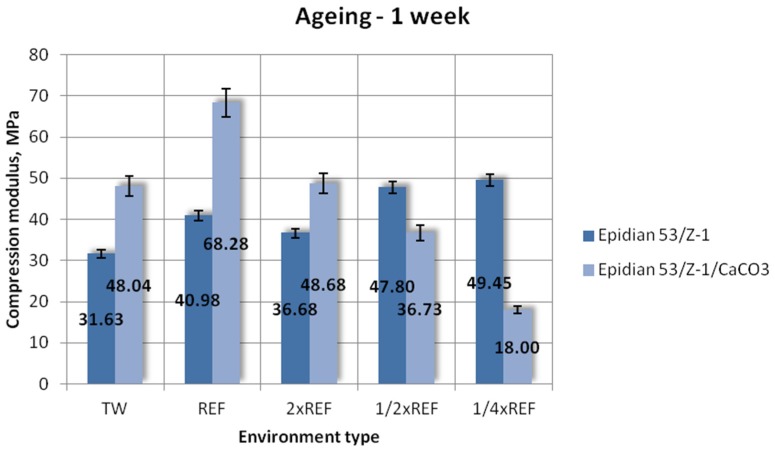
Compression modulus of the unmodified (Epidian 53/Z-1) and modified (Epidian 53/Z-1/CaCO_3_) epoxy adhesive compounds, aged for 1 week.

**Figure 7:**
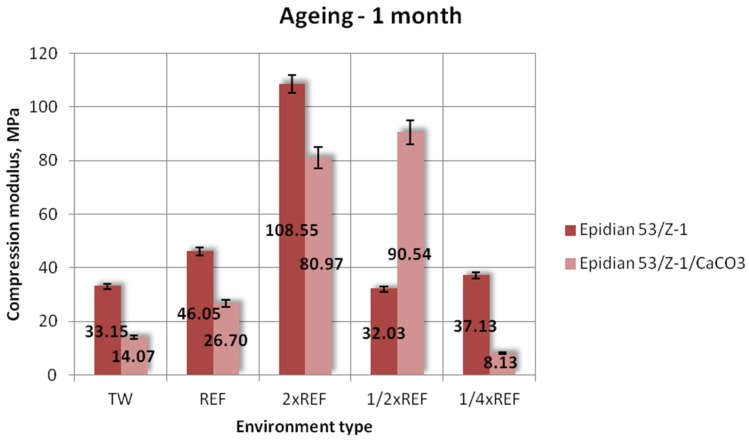
Compression modulus of the unmodified (Epidian 53/Z-1) and modified (Epidian 53/Z-1/CaCO_3_) epoxy adhesive compounds, aged for 1 month.

**Figure 8:**
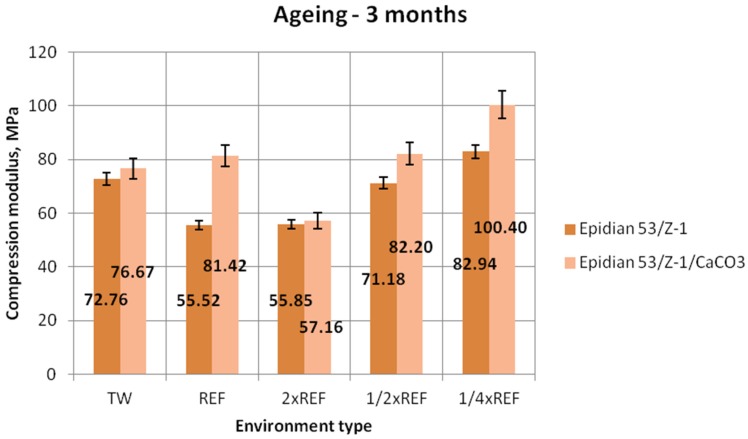
Compression modulus of the unmodified (Epidian 53/Z-1) and modified (Epidian 53/Z-1/CaCO_3_) epoxy adhesive compounds, aged for 3 months.

Figure 6, Figure 7 and Figure 8 show the results of compression modulus (mean values) obtained for the two tested types of epoxy adhesive compounds: one filled with calcium carbonate and the other without a filler, that were aged for one week, one month and three months, in different salt water environments.

The above results (Figure 6) demonstrate that the filler-modified epoxy adhesive compound has a much higher compression modulus than the unmodified compound. The highest compression modulus of 68.28 MPa was obtained for the Epidian 53/Z-1/CaCO_3_ compound aged in the reference environment. A slightly lower compression modulus was obtained for the samples aged in tap water (48.04 MPa) and those aged in the environment with double reference salinity (48.68 MPa). The lowest compression modulus was obtained for the Epidian 53/Z-1/CaCO_3_ samples aged in the environment containing a quarter of the reference salinity (18.00 MPa), amounting to only 26% of the maximum value achieved by this adhesive compound. The compression modulus results of the other tested epoxy adhesive compound, Epidian 53/Z-1, are lower than those of the modified adhesive. The highest value of 49.45 MPa was achieved by the samples aged in the environment containing a quarter of the reference salinity. The compression modulus of the samples aged in the solution containing 1/2 reference salinity is 47.80 MPa. Regarding other tested environments, the results obtained for this adhesive compound are significantly lower. The samples aged in tap water have the lowest compression modulus of 31.63 MPa. The difference between the maximum and minimum achieved values exceeds 36%. The plot also reveals that the compression modulus of the Epidian 53/Z-1 adhesive compound decreases with increasing salt concentration. As for the other epoxy adhesive compound, it can be observed that a reduction in salt concentration leads to a lower compression modulus value.

The results obtained after one month of aging plotted in Figure 7 clearly demonstrate that the Epidian 53/Z-1 adhesive compound has the highest compression modulus value of 108.55 MPa, and it was obtained for the samples aged in the double salinity environment. The samples aged in other tested environments have much lower compression modulus; the lowest of them is 32.03 MPa (seawater environment with 1/2 reference salinity) and amounts to only 29% of the maximum achieved value. In contrast, the highest compression modulus obtained for the calcium carbonate-filled adhesive compound samples (90.54 MPa) is slightly lower than that of the other tested epoxy adhesive compound, and it was obtained for the samples aged in the solution containing 1/2 reference salinity. The compression modulus of the samples aged in the double reference salinity environment was slightly lower (80.97 MPa), whereas the values obtained for the samples aged in other seawater environments were significantly lower. Nevertheless, the lowest compression modulus value obtained for this adhesive compound was 5.13 MPa, and it was obtained for the samples aged in the seawater environment with a quarter of the reference salinity value. The difference between the highest and lowest compression modulus values of Epidian 53/Z-1/CaCO_3_ was over 94%. The results demonstrate that the Epidian 53/Z-1 adhesive compound has a higher compression modulus.

The results of the Epidian 53/Z-1 adhesive compound (Figure 8) demonstrate that the highest compression modulus was obtained for the samples aged in the environment with a quarter of the reference salinity value (82.94 MPa). The lowest value (55.52 MPa) was achieved by the samples aged in the reference seawater environment. The difference between the maximum and minimum achieved compression moduli is about 33%. In the case of the modified adhesive compound (Epidian 53/Z-1/CaCO_3_), the highest compression modulus (100.40 MPa) was also obtained for the samples aged in the seawater environment with a quarter of the reference salinity value. The lowest value of 57.16 MPa was obtained for the samples aged in the solution with double reference salinity, and it amounted to 57% of the maximum value achieved for this adhesive compound. The samples aged in two other environments achieved almost the same compression modulus value, amounting to about 82% of the maximum value. Comparing the results of both adhesive compounds it can be observed that higher compression modulus values were obtained for the adhesive compound filled with calcium carbonate (CaCO_3_).

#### 3.1.3. Compressive Strain

**Figure 9:**
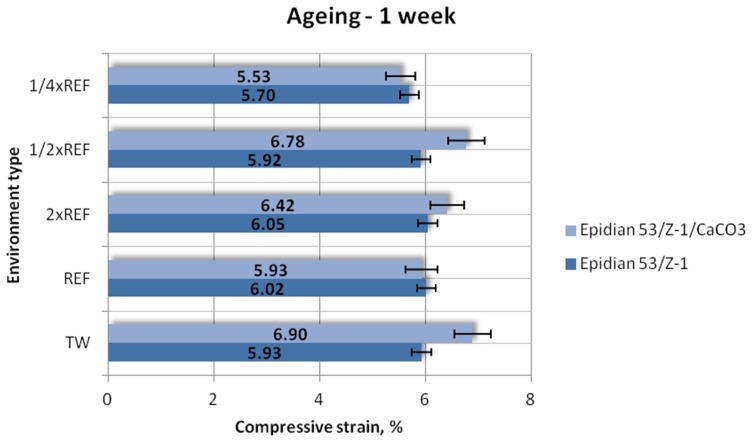
Compressive strain of the unmodified (Epidian 53/Z-1) and modified (Epidian 53/Z-1/CaCO_3_) epoxy adhesive compounds, aged for 1 week.

**Figure 10:**
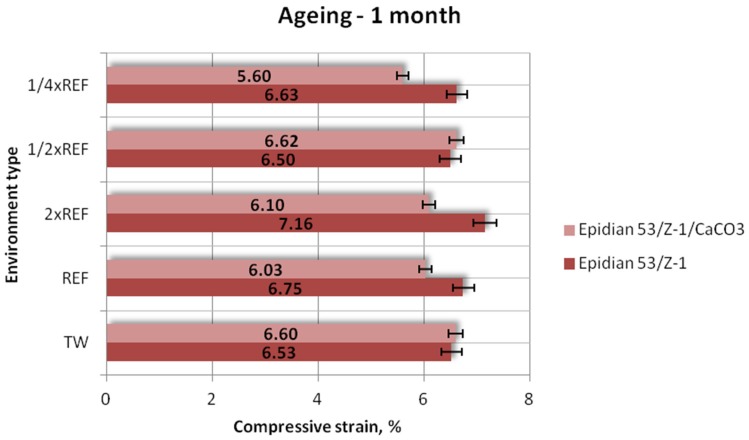
Compressive strain of the unmodified (Epidian 53/Z-1) and modified (Epidian 53/Z-1/CaCO_3_) epoxy adhesive compounds, aged for 1 month.

**Figure 11:**
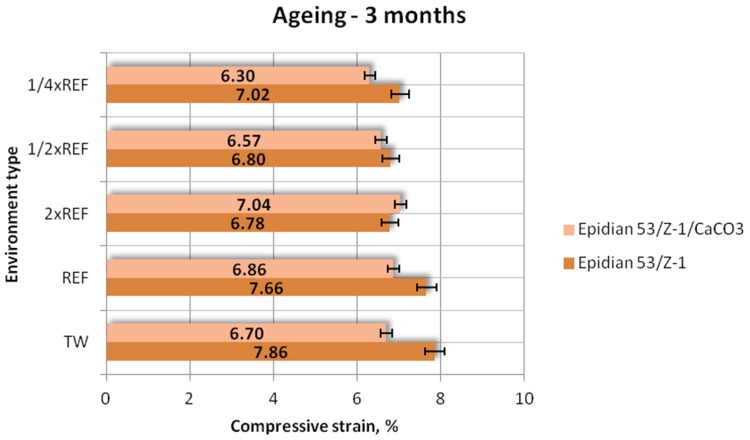
Compressive strain of the unmodified (Epidian 53/Z-1) and modified (Epidian 53/Z-1/CaCO_3_) epoxy adhesive compounds, aged for 3 months.

Results of the compressive strain (mean values) of the two tested types of adhesive compounds: unmodified and calcium-carbonate-filled, aged for one week, one month and one month in different seawater environments, are presented in Figure 9, Figure 10 and Figure 11.

The strength test results (Figure 9) of both epoxy adhesive compounds demonstrate that the Epidian 53/Z-1/CaCO_3_ samples have higher compressive strains. The highest compressive strain value of 6.90% was obtained for the samples aged in tap water. The lowest value (5.53%) was achieved by the samples aged in the solution containing a quarter of the reference salinity value. The difference between these values is 20%. Lower compressive strains were obtained for the unmodified adhesive compound. The highest compressive strain (6.05%) was achieved by the samples aged in the solution with double reference salinity. The samples aged in the solution containing a quarter of the reference salinity have the lowest compressive strain (5.70%). Nevertheless, this lowest value amounts to 94% of the maximum achieved value. The above results demonstrate that the compressive strain of the Epidian 53/Z-1/CaCO_3_ adhesive compound increases with the increasing salt concentration.

The above results (Figure 10) indicate differences between the compressive strains of the studied adhesive compounds aged for one month in the tested environments. Similar compressive strain values were obtained for the samples of Epidian 53/Z-1 and Epidian 53/Z-1/CaCO_3_ that were aged in two particular environments: tap water and 1/2 reference salinity solution. These values differ by only 1% in these environments. However, higher compressive strains were achieved by the samples of Epidian 53/Z-1. It is also worth noting to some extent that salt concentration affects the compressive strain of the samples aged for one month.

The results demonstrate that the unmodified adhesive compound has higher compressive strains (Figure 11). The highest values were obtained for the samples aged in tap water (7.86%) and the reference seawater (7.66%). The lowest compressive strain was obtained by the samples aged in the solution with double reference salinity (6.78%), which amounts to 86% of the maximum achieved compressive strain. Regarding the epoxy adhesive compound filled with CaCO_3_, the worst result was obtained for the samples aged in the environment with 1/4 reference salinity, with the compressive strain being equal to 6.30%, which is 89% of the maximum value achieved by Epidian 53/Z-1/CaCO_3_, i.e., 7.04 MPa. The above results indicate that the unmodified adhesive compound exhibits a greater difference between the values.

#### 3.1.4. Mechanical Properties of References Samples

Table 5 presents the mechanical properties: compressive strength, compression modulus and compressive strain of the reference samples of Epidian 53/Z-1 and Epidian 53/Z-1/CaCO_3_.

The above results (Table 5) demonstrate that the studied epoxy adhesive compounds have very similar compressive strengths. The compressive strength of Epidian 53/Z-1 is 71.06 MPa, while that of Epidian 53/Z-1/CaCO_3_ is equal to 72.22 MPa. The difference between these values is about 2%. Comparing the results of the reference samples with those obtained for the samples aged in different salt water environments, it can be observed that salt concentration affects the compressive strength of Epidian 53/Z-1. The results presented in Table 5 demonstrate that the Epidian 53/Z-1/CaCO_3_ adhesive compound has a much higher compressive modulus (74.52 MPa) than the unmodified adhesive compound. The compression modulus value is higher by over 43%, as it can be seen in the figure. Comparing the results of the reference samples and those of the samples aged in different seawater environments, it can be observed that Epidian 53/Z-1/CaCO_3_ has also significantly higher compression modulus values than the reference samples. The compressive strain of Epidian 53/Z-1 (6.40%) is higher than that of the calcium-carbonate-filled adhesive compound (5.52%), the difference between these values being equal to 13%. The Epidian 53/Z-1 adhesive compound samples, both the reference samples and those aged in different salt water environments, have higher compressive strains than the calcium-carbonate-modified adhesive compound. This means that the salt concentration does not affect this parameter to any significant extent.

### 3.2. Microscopic Results

#### 3.2.1. Reference Samples

**Figure 12:**
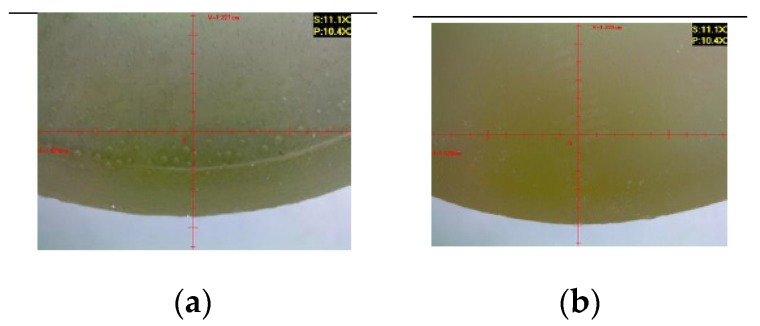
Reference samples: (**a**) Epidian 53/Z-1; (**b**) Epidian 53/Z-1/CaCO_3_.

Figure 12 presents the microscopic examination results obtained for the reference samples of Epidian 53/Z-1 and Epidian 53/Z-1/CaCO_3_.

Microscopic images of the reference samples serve as a basis for the comparison of the results of the modified and unmodified adhesive compound samples after aging (seasoning) in different salt water environments.

#### 3.2.2. Tap Water

**Figure 13:**
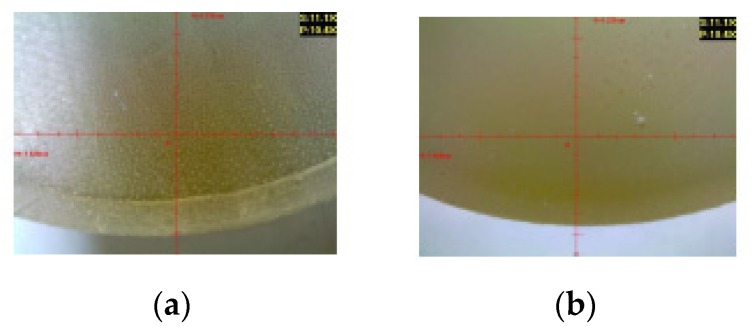
Samples aged in tap water for 3 months: (**a**) Epidian 53/Z-1; (**b**) Epidian 53/Z-1/CaCO_3_.

Figure 13 presents the microscopic examination results of examples of the two tested adhesive compounds after aging in tap water for three months (the longest analyzed aging time).

Based on the obtained microscopic images, it can be concluded that tap water has no effect on the samples of both adhesive compounds. No changes or inclusions in the form of sea salt sediment can be observed on the surface of the cylindrical samples.

#### 3.2.3. Seawater Environment (Reference Salinity)

Table 6 shows the results of microscopic examination of the samples of Epidian 53/Z-1 and Epidian 53/Z-1/CaCO_3_ adhesive compounds, aged in the reference seawater environment.

The above results demonstrate that the samples of both tested adhesive compounds aged for three months and one month have developed light gray sediment on their surface, which results from the salt content in the solution. In contrast, the samples aged in the reference environment for one week do not show any changes on their surface.

#### 3.2.4. Environment with Double Reference Salinity

Below is a comparison of the microscopic examination results (Table 7) of the cylindrical samples of Epidian 53/Z-1 and Epidian 53/Z-1/CaCO_3_ that were aged in the solution with double reference salinity.

The microscopic images given in Table 7 show changes on the surface of the Epidian 53/Z-1 sample aged for three months. Specifically, it can be observed that a sea salt sediment is deposited on the sample surface and a more extensive change resembling a corrosion pit is visible. The white color of this modification is stronger, which results from the deposition of a greater amount of salt. Despite the changes on the sample surface, the hardness of this sample did not change. The sample aged for one month is also slightly changed. A sediment is formed on its surface. The sediment, which results from the salt concentration in the solution, is considerably more visible in some parts of the sample surface. Nevertheless, the sample did not undergo any significant changes. The sample aged for one week has a small amount of sea salt sediment on its surface. Apart from that, no other changes can be observed. In contrast, the Epidian 53/Z-1/CaCO_3_ samples aged in different environments for the three tested aging times show no changes on their surface at all.

#### 3.2.5. Environment with 1/2 Reference Salinity

Table 8 presents the microscopic examination results of cylindrical samples subjected to aging in the environment with 1/2 reference salinity. The samples were made of Epidian 53/Z-1 and Epidian 53/Z-1/CaCO_3_ adhesive compounds.

The microscopic images in Table 8 show the samples of both adhesives after aging in the solution with 1/2 reference salinity for three different periods. The Epidian 53/Z-1 sample aged for three months has been significantly affected by salt water, in particular on its edge. This is manifested as the delamination of the adhesive compound edge, considerable softening of the material and a white-color sea salt sediment. The Epidian 53/Z-1/CaCO_3_ sample aged for three months also shows the presence of the significant changes on its surface depending on the type of tested environment. Compared to the previous sample, however, these changes are less extensive. The surface of the sample has become white, which is typical of the deposited sea salt, and there are small changes on the sample’s edge. In this case, the changes are more substantial on the surface of the tested sample than on its edge. In contrast, no changes have been observed for the samples of both adhesive compounds aged for one month and one week.

#### 3.2.6. Environment with 1/4 Reference Salinity

Results of the microscopic examination are given in Table 9. The epoxy adhesive samples of Epidian 53/Z-1 and Epidian 53/Z-1/CaCO_3_ were aged in the environment with a quarter of the reference salinity value.

Table 9 presents the results obtained for the samples aged in the environment containing a quarter of the reference salinity value. After three months of aging, the surface of the Epidian 53/Z-1 sample has developed a visible, irregularly shaped modification on its surface, around which small mass losses and sea salt sediment are visible. The sample surface in the changed area is softer than the surface of the reference sample made of the same adhesive compound. Some changes can also be observed on the surface of the Epidian 53/Z-1/CaCO_3_ sample. Nevertheless, these changes are less profound than those observed for the previous sample. The surface of the sample is of a light reddish-brown color and is covered with the sea salt sediment. The formation of the corrosion-resembling defect could be caused by the presence of metal filings from the samples in the solution, because, following their removal from the cylindrical molds, the samples were made even with the use of a saw and sandpaper. The microscopic examination shows that other samples aged in this environment did not undergo any changes.

## 4. Discussion

The epoxy resin is in the form of a long molecular chain with reactive sites at both ends. Reactive sites are formed by epoxy groups, and the lack of ester groups means that the epoxy-based polymer chain has excellent water resistance (as well as chemical agents). The name “epoxy” refers to a chemical group consisting of an oxygen atom connected to two carbon atoms that are bonded to each other. The epoxide molecule has also two ring groups in the center that withstand mechanical and thermal stresses much better than linear groups, thanks to which the epoxies have very good strength, stiffness and thermal properties. An important property of any resin, especially in the marine environment, is resistance to degradation under the influence of water. All resins retain some moisture, which increases the mass of the cured material. With the tested aging time, the water absorption of the epoxy composition increases. According to Lettieri and Frigione [31], the water ingress led to the plasticization of the adhesive, enhanced the reactivation of crosslinking reactions, as well as the erasure of physical aging. Epoxy resins are not as susceptible to degradation under the influence of water compared to other resins due to the lack of hydrolysis sensitive ester groups in their molecular structure. For example, an epoxy laminate will retain approximately 90% interlayer shear strength after being immersed in water for a year, while a polyester laminate, for example, will retain approximately 65%. This is due to the fact that polyester (including vinyl ester) resins are susceptible to degradation under the influence of water due to the presence in their molecular structure of ester groups sensitive to hydrolysis.

Summing up the results of strength tests and microscopic tests, it can be seen that:

- The mechanical properties of the studied epoxy adhesive compounds depend on the type of adhesive, aging time and tested environment;
- The highest compression modulus was obtained for the samples of Epidian 53/Z-1/CaCO_3_ aged for three months in the environment containing a quarter of the reference salinity value;
- The samples of Epidian 53/Z-1 have the highest compression modulus value after one-month aging in the solution containing double reference salinity;
- The samples of Epidian 53/Z-1/CaCO_3_ have the highest compression modulus values after aging for three months and one week;
- In most cases, the samples of Epidian 53/Z-1 aged in salt water for one month have higher compression modulus values;
- The compression modulus of the reference samples of Epidian 53/Z-1/CaCO_3_ is 43% higher than that of the unmodified adhesive compound;
- The highest compressive strength was obtained for the Epidian 53/Z-1/CaCO_3_ samples that were aged for three months in double reference salinity solution;
- The samples of Epidian 53/Z-1 aged for three months in the solution containing a quarter of the reference salinity value have the highest compressive strength;
- The samples of Epidian 53/Z-1 aged for one month and one week have considerably higher compressive strengths;
- The samples of Epidian 53/Z-1/CaCO_3_ aged for three months have the highest compressive strength;
- The reference samples of both epoxy adhesive compounds have almost identical compressive strengths;
- The highest compressive strains were obtained for the samples of Epidian 53/Z-1/CaCO_3_ aged for three months in the solution with double reference salinity;
- The highest compressive strain value was obtained for the Epidian 53/Z-1 samples aged in tap water for three months;
- The highest compressive strains were obtained for the Epidian 53/Z-1 samples aged for three months and one week;
- The samples of Epidian 53/Z-1/CaCO_3_ aged for one week have a higher compressive strain;
- The compressive strain of the reference samples of Epidian 53/Z-1 is only 13% higher than that of the Epidian 53/Z-1/CaCO_3_ samples;
- The microscopic examination has shown that the samples aged in salt water for three months undergo more changes;
- The cylindrical samples of Epidian 53/Z-1 aged in the solution with double reference salinity for three months and one month undergo only small changes.

The results demonstrate that the compressive strength of the reference samples (not subjected to aging in tap and salt water environments) is lower than that of the Epidian 53/Z1 adhesive samples aged in salt water. It should be noted, however, that in some cases, in the first phase of degradation, the degradation agent improves certain material properties, especially mechanical strength. This is done by additional crosslinking of the material structure under the influence of, for example, heat. It is only at a later stage that other processes become apparent, e.g., excessive crosslinking or molecular weight reduction, which causes the tested properties to deteriorate. Fernades et al. [13] presented that in the salt water environment, the increase in fracture toughness can be explained as a result of the interaction between two opposite factors. Differently, the absorbed water causes a slight degradation (glass transition temperature was reduced). On the other hand, this degradation is not enough to overcome the increased ductility caused by the plasticization of the adhesive. Heshamti et al. [15] investigated the effects of aging adhesive and bonded joints in five harsh environments, including salt water solution, at various temperatures (20 °C and 45 °C). The results reported that, among other things, the tensile strength was significantly degraded with the increased exposure duration and the severity of the aging condition. Uthaman et al. [32] also underlined that degradation adversely affected the tensile strength, although the tensile modulus values did not significantly decrease throughout the aging study. In the study presented by Heshamati et al. [9], the aging time was set to 210 and 840 days, whereas in the present study the longest aging time was 90 days. Perhaps a longer aging period would have caused more significant changes. In the studied case, the longest immersion time in water was three months, therefore no significant trend of reduction in strength parameters was observed, but rather the increase in selected parameters. Probably a further increase in immersion time would reduce these parameters. Moreover, the study described in this paper also involved investigating compressive strength. According to Heshmati et al. [9], salt water degrades the mechanical properties of adhesives to a lesser degree than distilled water. A similar trend was observed in the present study despite the fact that salt water was compared to tap water. Narynbek Ulu et al. [27] underlined that the damage mechanisms (in the case of elastomers) in seawater became complex and not completely understood. Further research on this subject is planned, because it is an interesting issue, especially in the field of using laminates and epoxy composites in the construction of yachts, as well as protective covers for vessels, which was also emphasized in Nash et al. [33] work. The authors investigated an infusible thermoplastic matrix system compared to matrix materials most commonly used in marine structures under various immersion conditions and developed the procedure to indicate the suitable materials to marine construction vessels.

## 5. Conclusions

Summing up, it can be stated that the mechanical properties of Epidian 53/Z-1 and Epidian 53/Z-1/CaCO_3_ epoxy adhesive compounds are affected by different factors. The results have shown that these properties depend on the type of adhesive compound and water environment. The experiments have determined which of the tested epoxy adhesive compounds has the highest mechanical properties. The Epidian 53/Z-1 adhesive compound achieved better results for most strength parameters. Nevertheless, the strength test results of the epoxy adhesive filled with calcium carbonate (CaCO_3_) are also considerably high. Both tested epoxy adhesive compounds obtained high strength results, depending on the seawater environments and aging time. The Epidian 53/Z-1/CaCO_3_ epoxy adhesive compound has the highest compression modulus; the highest values were obtained for the reference samples and those aged in salt water for three months and one week. The compressive strength of the Epidian 53/Z-1 samples increased after aging in salt water for one month and one week. This adhesive compound also has a higher compressive strain. Compared to the epoxy adhesive compound filled with calcium carbonate (CaCO_3_), the samples of Epidian 53/Z-1 aged for three months and one month have higher compression modulus values. These points to the importance of tailoring the selection of an adhesive compound to the environment in which it will be used.

## Figures and Tables

**Table 1:** Mechanical properties of cured Epidian 53 epoxy resin [28].

Properties	Value
Bending strength (MPa)	80–100
Compressive strength (MPa)	70–90
Shear strength of joint cured for 16 h at 20–25 °C, 6 h at 80 ± 2 °C (MPa), not lower than	7.84
Shear strength of joint cured for 7 days at 20–25 °C (MPa), not lower than	5.86

**Table 2:** Properties of triethylenetetramine curing agent [28].

Properties	Value
Viscosity, 25 °C (mPa·s)	20–30
Density, 20 °C (g/cm^3^)	0.98
Amino number (mgKOH/g)	min. 1100

**Table 3:** Tested epoxy adhesive compounds.

Characteristics of Epoxy Adhesive Compound Components		
Variant of epoxy adhesive compounds	Unmodified	Modified
Resin type	Epidian 53	Epidian 53
Amount of resin, g	100	100
Curing agent type	Amine (triethylenetetramine)	Amine (triethylenetetramine)
Amount of curing agent, g	80	80
Filler type	-	Calcium carbonate (CaCO_3_)
Amount of filler, g	-	2
Epoxy compound designation	Epidian 53/Z-1	Epidian 53/Z-1/CaCO_3_

**Table 4:** Tested environments with different salt concentrations.

Type of Environment	Salt Concentration	Symbol
Tap water	-	TW
Seawater (reference)	35 g NaCl/1000 g tap water	REF
Seawater with 2× reference salinity	70 g NaCl/1000 g tap water	2× REF
Seawater with 1/2 reference salinity	17.5 g NaCl/1000 g tap water	1/2× REF
Seawater with 1/3 reference salinity	8.8 g NaCl/1000 g tap water	1/4× REF

**Table 5:** Compressive strength of the reference samples of Epidian 53/Z-1 and Epidian 53/Z-1/CaCO_3_.

Mechanical Properties	Adhesive Type	
	Epidian 53/Z-1	Epidian 53/Z-1/CaCO_3_
Compressive strength, MPa	71.06	72.22
Compression modulus, MPa	42.22	74.52
Compressive strain, %	6.40	5.52

**Table 6:** Samples aged in the reference seawater environment.

Epidian 53/Z-1	Epidian 53/Z-1/CaCO_3_
Aged time: 3 months	
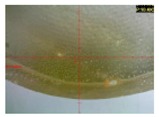	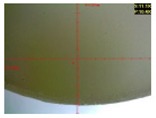
Aged time: 1 month	
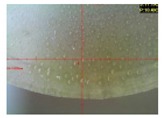	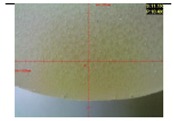
Aged time: 1 week	
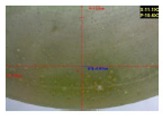	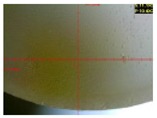

**Table 7:** Samples aged in double reference salinity environment.

Epidian 53/Z-1	Epidian 53/Z-1/CaCO_3_
Aged time: 3 months	
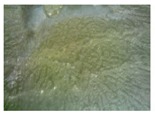	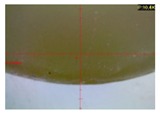
Aged time: 1 month	
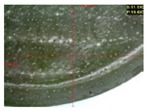	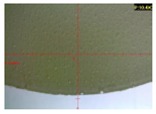
Aged time: 1 week	
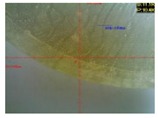	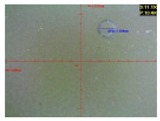

**Table 8:** Samples aged in 1/2 reference salinity environment.

Epidian 53/Z-1	Epidian 53/Z-1/CaCO_3_
Aged time: 3 months	
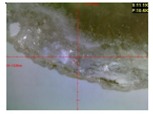	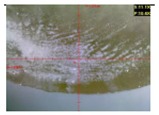
Aged time: 1 month	
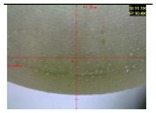	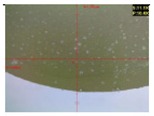
Aged time: 1 week	
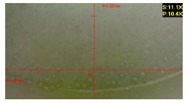	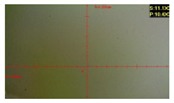

**Table 9:** Samples aged in 1/4 reference salinity environment.

Epidian 53/Z-1	Epidian 53/Z-1/CaCO_3_
Aged time: 3 months	
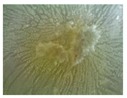	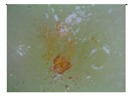
Aged time: 1 month	
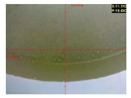	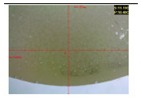
Aged time: 1 week	
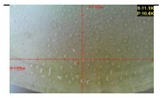	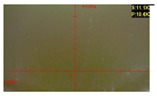

## References

[1] Pizzi A., Mittal K.Z. (2018). Handbook of Adhesive Technology.

[2] Lee H.L., Neville H. (1988). Handbook of Epoxy Resins.

[3] Rudawska A., Pizzi A., Mittal K.Z. (2018). Epoxy adhesives. Handbook of Adhesive Technology.

[4] Pertie E.M. (2006). Epoxy Adhesive Formulation.

[5] Pethrick R.A. (2015). Design and ageing of adhesives for structural adhesive bonding—A review. J. Mater. Design Appl..

[6] Rudawska A., Brunella V. (2020). The effect of ageing in water solution containing iron sulphate on the mechanical properties of epoxy adhesives. Polymers.

[7] Rudawska A. (2019). The impact of seasoning conditions on mechanical properties of modified and unmodified epoxy adhesive compounds. Polymers.

[8] Sugiman S., Crocombe A.D., Aschroft I.A. (2013). The fatigue response of environmentally degraded adhesively bonded aluminium structures. Int. J. Adhes. Adhes..

[9] Heshmati M., Haghani R., Al-Emrani M. (2017). Durability of CFRP/steel joints under cyclic wet-dry and freeze-thaw conditions. Compos. B.

[10] Leger R., Roy A., Grandidier J.C. (2013). A study of the impact of humid aging on the strength of industrial adhesive joints. Int. J. Adhes. Adhes..

[11] Viana G., Costa M., Banea M.D., da Silva L.F.M. (2017). Water diffusion in double cantilever beam adhesive joints. Latin Am. J. Solid Struct..

[12] De Neve B., Shanahan M.E.R. (1995). Physical and chemical effects in an epoxy resin exposed to water vapour. J. Adhes..

[13] Fernades P., Viana G., Carbas R.J.C., Costa M., da Silva L.F.M., Banea M.D. (2017). The influence of water on the fracture envelope of an adhesive joint. Theor. Appl. Fract. Mech..

[14] Lai M., Botsis J., Cugnoni J., Coric D. (2012). An experimental-numerical study of moisture absorption in an epoxy. Compos. A.

[15] Heshmati M., Haghani R., Al-Emrani M. (2016). Effects of moisture on the long-term performance of adhesively bonded FRP/steel joints used in bridges. Compos. B.

[16] Sugiman S., Crocombe A.D., Aschroft I.A. (2013). Experimental and numerical investigation of the static response of environmentally aged adhesively bonded joints. Int. J. Adhes. Adhes..

[17] Lefebvre D.R., Takahashi K.M., Muller A.J., Raju V.R. (1991). Degradation of epoxy coatings in humid environments: The critical relative humidity for adhesion loss. J. Adhes. Sci. Technol..

[18] Lefebvre D.R., Elliker P.R., Takahashi K.M., Raju V.R., Kaplan M.L. (2000). The critical humidity effect in the adhesion of epoxy to glass: Role of hydrogen bonding. J. Adhes. Sci. Technol..

[19] Leger R., Roy A., Grandidier J.C. (2010). Non-classical water diffusion in an industrial adhesive. Int. J. Adhes. Adhes..

[20] Blackburn B.P., Tatar J., Douglas E.P., Hamilton H.R. (2015). Effect of hydrothermal conditioning on epoxy adhesives used in FRP composites. Constr. Build. Mater..

[21] Bordes M., Davies P., Cognard J.-Y., Sohier L., Sauvant-Moynot V., Galy J. (2009). Prediction of long term strength of adhesively bonded steel/epoxy joints in sea water. Int. J. Adhes. Adhes..

[22] Fiore V., Calabrese L., Proverbio E., Galtieri G., Scalici T., Lo Presti V.M. (2016). Pull-off adhesion of hybrid glass-steel adhesive joints in salt fog environments. J. Adhes. Sci. Technol..

[23] Hua D., Lin J., Zhang B. (2013). Effects of salt spray on the mechanical properties of aluminium-epoxy adhesive joints. J. Adhes. Sci. Technol..

[24] Gao Y.-L., Xiong J.-P., Zhang S., Zuo Y. (2006). Aging performance of epoxy adhesive in salt water. Adv. Mater. Res..

[25] Wang C., Huang Y.D., Xv H.Y., Liu W.B. (2004). The durability of adhesive/carbon-carbon composites joints in salt water. Int. J. Adhes. Adhes..

[26] Wen S.W., Xiao J.Y., Wang Y.R. (2013). Accelerated aging behaviors of aluminium plate with composite patches under salt fog effect. Compos. B.

[27] Narynbek Ulu K., Huneau B., Le Gac P.-Y., Verron E. (2016). Fatigue resistance of natural rubber in seawater with comparison to air. Int. J. Adhes. Adhes..

[28] Ciech Resins. http://www.ciechzywice.pl/pl/produkty/chemia-organiczna/zywice/zywice-epoksydowe.

[29] Miturska I., Rudawska A., Müller M., Valášek P. (2020). The Influence of Modification with Natural Fillers on the Mechanical Properties of Epoxy Adhesive Compositions after Storage Time. Materials.

[30] Salinity. https://en.wikipedia.org/wiki/Salinity.

[31] Lettieri M., Frigione M. (2012). Effects of humid environment on thermal and mechanical properties of a cold-curing structural epoxy adhesive. Constr. Build. Mater..

[32] Uthan A., Xian G., Thomas S., Wang Y., Zheng Q., Liu X. (2020). Durability of an epoxy resin and its carbon fiber-reinforced polymer composite upon immersion in water, acidic, and alkaline solutions. Polymers.

[33] Nash N.H., Portela A., Bachour-Sirerol C.I., Manolakis I., Comer A.J. (2019). Effect of environmental conditioning on the properties of thermosetting-and thermoplastic-matrix composite materials by resin infusion for marine applications. Compos. B.

